# A Method to Study the Distribution Patterns for Metabolites in Xylem and Phloem of Spatholobi Caulis

**DOI:** 10.3390/molecules25010167

**Published:** 2019-12-31

**Authors:** Yuqi Mei, Lifang Wei, Chuan Chai, Lisi Zou, Xunhong Liu, Jiali Chen, Mengxia Tan, Chengcheng Wang, Zhichen Cai, Furong Zhang, Shengxin Yin

**Affiliations:** College of Pharmacy, Nanjing University of Chinese Medicine, Nanjing 210023, China; 18260028173@163.com (Y.M.); weilifangquiet@163.com (L.W.); echo_0523@hotmail.com (C.C.); zlstcm@126.com (L.Z.); 18994986833@163.com (J.C.); 18816250751@163.com (M.T.); ccw199192@163.com (C.W.); caizhichen2008@126.com (Z.C.); frfr123@126.com (F.Z.); yinshengxin723@163.com (S.Y.)

**Keywords:** Spatholobi Caulis, xylem, phloem, metabolites, distribution patterns

## Abstract

Spatholobi Caulis (SC), the vine stem of *Spatholobus suberectus* Dunn, is a widely used traditional Chinese medicine (TCM) for the treatment of blood stasis syndrome and related diseases. Xylem and phloem are the main structures of SC and the color of xylem in SC is red brown or brown while the phloem with resin secretions is reddish brown to dark brown. They are alternately arranged in a plurality of concentric or eccentric rings. In order to investigate the distribution patterns of metabolites in xylem and phloem of SC, an analytical method based on UFLC–QTRAP–MS/MS was established for simultaneous determination of 22 constituents including four flavanols, nine isoflavones, two flavonols, two dihydroflavones, one flavanonol, one chalcone, one pterocarpan, one anthocyanidin and one phenolic acid in the samples (xylem and phloem) from Laos. Furthermore, according to the contents of 22 constituents, heat map, principal components analysis (PCA), orthogonal partial least squares discriminant analysis (OPLS–DA) and t–test were used to evaluate the samples and discover the differences between xylem and phloem of SC. The results indicated that the measured ingredients in xylem and phloem were significantly different. To be specific, the contents of flavonoids in xylem were higher than that in phloem, while the content of protocatechuic acid showed a contrary tendency. This study will not only reveal the distribution patterns of metabolites in xylem and phloem of SC but also facilitate further study on their quality formation.

## 1. Introduction

Spatholobi Caulis (SC), the vine stem of *Spatholobus suberectus* Dunn of the family Leguminosae, is specified in Chinese Pharmacopoeia (2015 version) with the name of Jixueteng. SC has been mainly prescribed for the treatment of irregular menstruation, dysmenorrhea, amenorrhea, rheumatism, blood deficiency and chlorosis [1]. Modern phytochemical investigations have revealed that SC contains diverse secondary metabolites, such as flavonoids, terpenoids, sterols, anthraquinones and organic acids [2]. Among these compounds, the flavonoids possess various pharmacological activities including anti-tumor [3,4,5], anti-virus [6], anti-oxidation [7] and anti-anemia [8]. In addition, protocatechuic acid has antibiosis and anti-inflammatory effects and accounts significant pharmacological activity for the cardiovascular system [9]. Xylem and phloem are the main structures of SC and the color of xylem in SC is red brown or brown while the phloem with resin secretions is reddish brown to dark brown. They are alternately arranged in a plurality of concentric or eccentric rings [1,10,11]. Traditional Chinese medicine (TCM) is a complex system composed of many chemical components, whose quality is closely related to the metabolites [12]. In recent years, there have been a few studies regarding the quantification of the active constituents in SC [13,14,15,16]. However, little attention was paid to the distribution of metabolites in different tissues of SC. Therefore, it is necessary to develop a rapid and reliable method to explore the distribution patterns of metabolites in xylem and phloem of SC with the hope to provide basis for the investigation of its quality formation.

In this study, ultra-fast performance liquid chromatography coupled with triple quadrupole–linear ion trap mass spectrometry (UFLC–QTRAP–MS/MS) was developed to simultaneously determine the contents of 22 constituents including 4 flavanols, 9 isoflavones, 2 flavonols, 2 dihydroflavones, 1 flavanonol, 1 chalcone, 1 pterocarpan, 1 anthocyanidin and 1 phenolic acid in the samples (xylem and phloem) from Laos. Furthermore, multivariate statistical analysis was applied to this study according to the 22 constituents tested. Principal component analysis (PCA) was used to get a good overview of the sample distribution. The samples were analyzed by orthogonal partial least squares discriminant analysis (OPLS–DA) to find out the differential constituents which were associated with the sample classifications. T–tests were performed to show the difference of each compound between the two sections of SC [17,18,19,20,21]. The proposed investigation could bring to light the distribution patterns of metabolites in xylem and phloem of SC and provide the basis for exploring the accumulation rule of metabolites in SC. In addition, this study will also have a certain reference value for studying the quality formation mechanism of SC.

## 2. Results

### 2.1. Optimization of Extraction Conditions

In this study, extraction variables such as extraction solvent (30%, 50%, 70% and 90% aqueous methanol; 30%, 50%, 70% and 90% aqueous ethanol; chloroform–methanol (4:1), *v*/*v*), extraction time (30, 60 and 90 min) and solid–liquid ratio (1:10, 1:20, 1:30 and 1:40, *v*/*v*) were optimized in order to obtain satisfactory extraction efficiency of Catechin, Epicatechin, Gallocatechin, Epigallocatechin and Formononetin by using the single factor test. The results showed that ultrasonic extraction with a 1:10 ratio of chloroform–methanol (4:1) for 60 min at room temperature were the conditions that allowed a higher yield of extraction of the target compounds.

### 2.2. Optimization of UFLC Conditions

In order to achieve the optimal elution conditions, single factor test was employed to investigate the types of column and mobile phase. In this experiment, three types of reverse phase columns including Thermo Acclaim^TM^ RSLC 120 C_18_ column (2.1 mm × 150 mm, 2.2 μm) (Thermo Scientific, Waltham, MA, USA), Synergi^TM^ Hydro–RP100Å column (2.0 mm × 100 mm, 2.5 μm) (Phenomenex, Los Angeles, CA, USA) and XBridge^®^ C_18_ column (4.6 mm × 100 mm, 3.5 μm) (Waters, Wexford, Ireland) were evaluated. As a result, better separation effect and detection sensitivity were achieved on a XBridge^®^ C_18_ column (4.6 mm × 100 mm, 3.5 μm). In addition, different kinds of mobile phases (water–methanol, water–acetonitrile, 0.1% (*v*/*v*) formic acid water solution–methanol solution, 0.1% (*v*/*v*) formic acid water solution–acetonitrile solution and 0.3% (*v*/*v*) formic acid water solution–methanol solution), flow rates (0.6, 0.7 and 0.8 mL/min) and column temperatures (25, 30, 35 °C) were examined and compared. Finally, the detected components showed good sensitivity and resolution when eluted with 0.3% (*v*/*v*) formic acid water solution–methanol solution at a flow rate of 0.8 mL/min under 30 °C.

### 2.3. Optimization of MS Conditions

**Figure 1:**
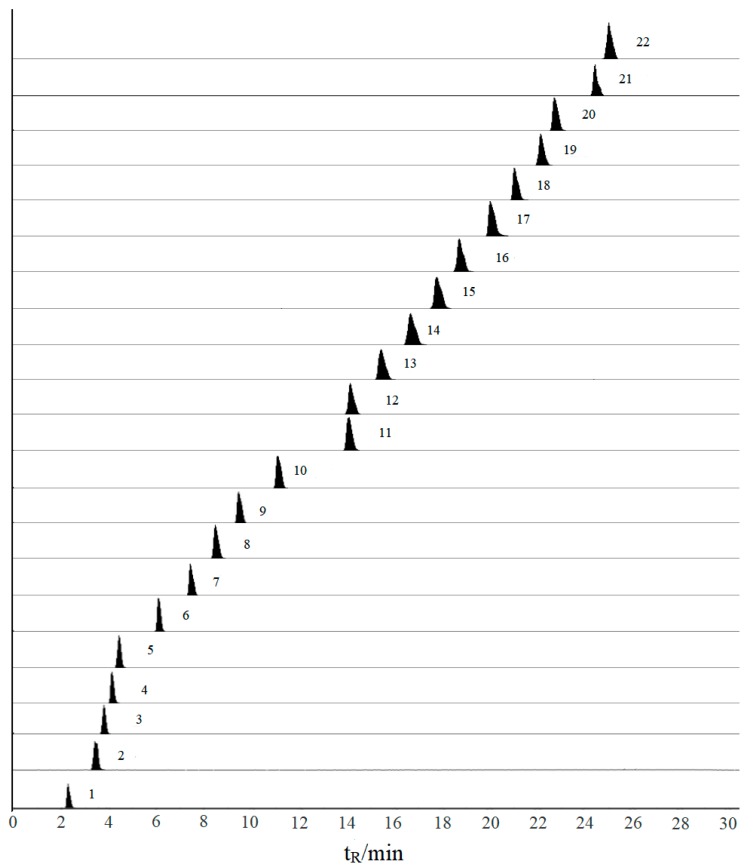
Representative extract ions chromatograms (XIC) of multi-reaction monitoring (MRM) chromatograms of 22 investigated compounds. (Compounds were shown in Table 1).

To develop a sensitive and precise quantitative method, individual solutions of all standards were detected by a full–scan mass spectrometry method in both positive and negative modes individually. After trial and error inspection, protocatechuic acid, kaempferol and genistin have good sensitivity and intensity in the negative ion mode. On the contrary, the other compounds have a good condition in the positive ion mode. Consequently, the electrospray ionization including ESI^+^ and ESI^–^ modes were simultaneously adopted in this study. Although the retention time of some compounds were similar, they could be accurately quantified based on different precursor and product ion pairs. For the optimization of multi-reaction monitoring (MRM) conditions, the parameters of declustering potential (DP) and collision energy (CE) were optimized to get the richest relative abundance of precursor and product ions. The optimum details, including retention time (t_R_), precursor and product ions, declustering potential (DP) and collision energy (CE) of 22 compounds, were listed in Table 1. MRM of 22 compounds were showed in Figure 1.

### 2.4. Method Validation

#### 2.4.1. Linearity, Limit of Detection and Limit of Quantification

All quantitative method validations were performed using the UFLC–QTRAP–MS/MS technology. Each standard calibration curve was obtained by plotting the peak area (Y) versus the corresponding concentration (X, ng/mL), which exhibited good linearity with appropriate correlation coefficients (*r*^2^ > 0.9990). The limits of detection (LODs) and limits of quantification (LOQs) of each analyte were measured at a signal/noise ratio of about 3 and 10, respectively, under the same chromatographic conditions. Results showed that the LODs and LOQs were in the range of 0.07–7.99 ng/mL and 0.22–26.63 ng/mL of 22 analytes, respectively. The results were shown in Table 2.

#### 2.4.2. Precision, Repeatability, Stability, Accuracy and Matrix Effect

The relative standard deviations (RSDs) of intra–day and inter–day variations of 22 components ranged from 0.93% to 4.20% and from 1.75% to 4.30%, respectively. The repeatability presented as RSDs were in the range of 0.76% to 4.42%. And the stability test of the 22 analytes presented as RSDs were between 0.73% and 4.24%. The overall recoveries varied from 95.88% to 103.93%, with RSDs < 4.94%, indicating that this method was validated for all analytes. The slope ratio values of the matrix curve to pure solution curve are between 0.93 and 1.08, indicating that the matrix had no significant effect on the ionization of analytes under the optimized conditions. The detailed results of each method validation were presented in Table 2.

### 2.5. Quantitative Analysis of Samples

**Figure 2:**
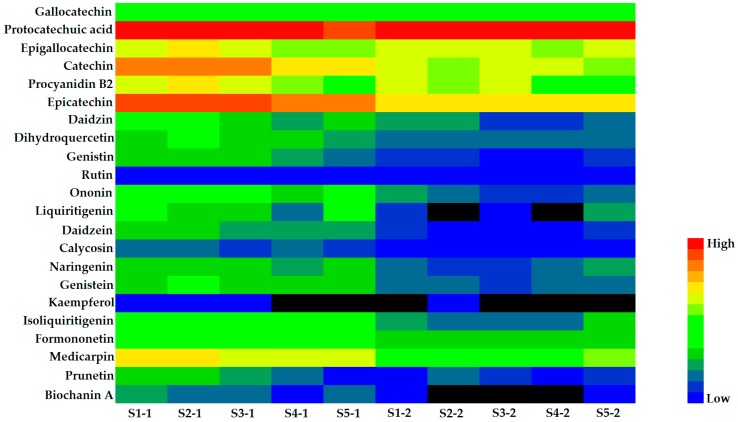
The content difference of 22 compounds by heat map.

The validated analytical method of UFLC–QTRAP–MS/MS was successfully applied to analyze 10 samples, including 5 batches of xylem and 5 batches of phloem of SC. A total of 22 components (4 flavanols, 9 isoflavones, 2 flavonols, 2 dihydroflavones, 1 flavanonol, 1 chalcone, 1 pterocarpan, 1 anthocyanidin and 1 phenolic acid) were quantified with a one point external standard method based on respective calibration curves. The quantitative results of 22 components were summarized in Table 3, indicating significant differences were shown in bioactive constituents between xylem and phloem of SC, as shown in Figure 2.

**Figure 3:**
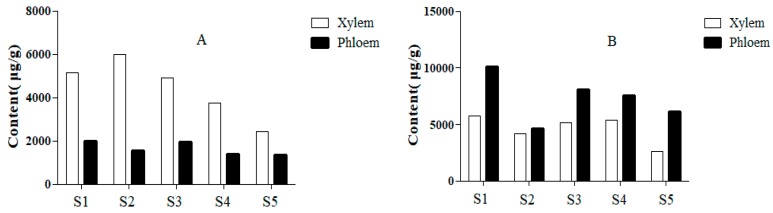
Contents of flavonoids (**A**) and protocatechuic acid (**B**) in xylem and phloem of SC.

The results manifested that protocatechuic acid (**2**) had a higher content, which accounted for about 66.08% of the total contents of all analytes tested in this study and the flavonoids component were the second, among which flavanols were the highest and in the following order: (highest) flavanols > pterocarpans > anthocyanidins > isoflavones > chalcones > dihydroflavones > flavanonols > flavonols (lowest). The 21 flavonoids were ranged from 0.00 to 2519.83 μg/g in xylem and from 0.00 to 808.65 μg/g in phloem. However, the contents of protocatechuic acid (**2**) were ranged from 2586.10 to 5745.66 μg/g and from 4607.29 to 10,118.61 μg/g in xylem and phloem, respectively. Combined with the results of metabolites detected, Figure 3 showed that the contents of flavonoids in xylem were higher than that in phloem. In stark contrast to this, the content of protocatechuic acid in xylem was lower than that in phloem. It is thus clear that the accumulation of flavonoids of SC was mostly distributed in the xylem, while the protocatechuic acid was mainly distributed in the phloem.

### 2.6. PCA of Samples

**Figure 4:**
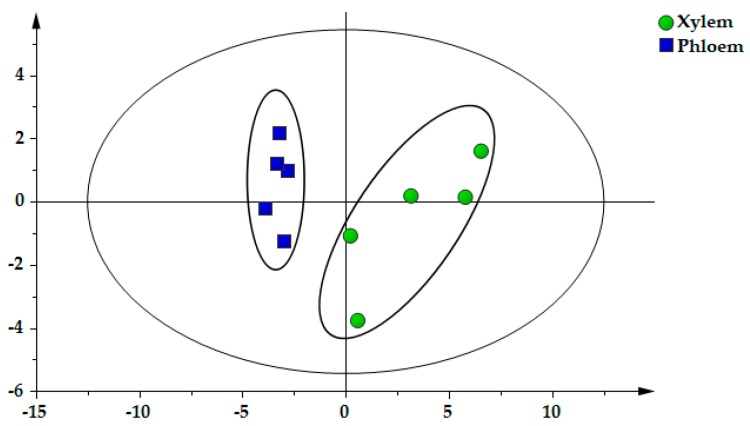
The principal component analysis (PCA) scores scatter plot of xylem and phloem in Spatholobi Caulis (SC).

In the light of the contents of 22 components, the xylem and phloem of SC were classified and distinguished by PCA. In the PCA scores plot (Figure 4), each point on the coordinate represents a sample and it could be seen that the defined samples were specifically separated into two clusters: xylem is mainly gathered in the positive axis of t [1], while phloem is mostly distributed on the negative axis of t [1]. The first two principal components (PC1 and PC2) occupied more than 80%, which could be used to represent overall information of samples (R^2^X [1] = 0.709, R^2^X [2] = 0.134). Thus, it can be seen that PCA may be an efficacious method to differentiate xylem and phloem by virtue of the distribution on the PC axis, which provided the basis for revealing the chemical distribution of SC.

### 2.7. OPLS–DA of Samples

**Figure 5:**
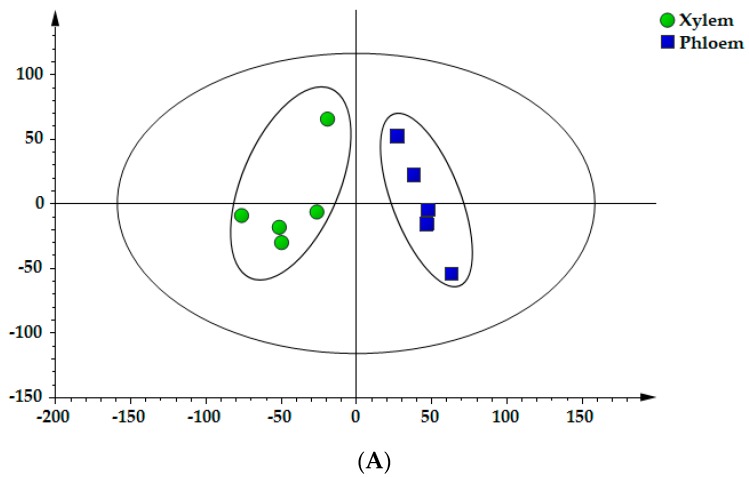

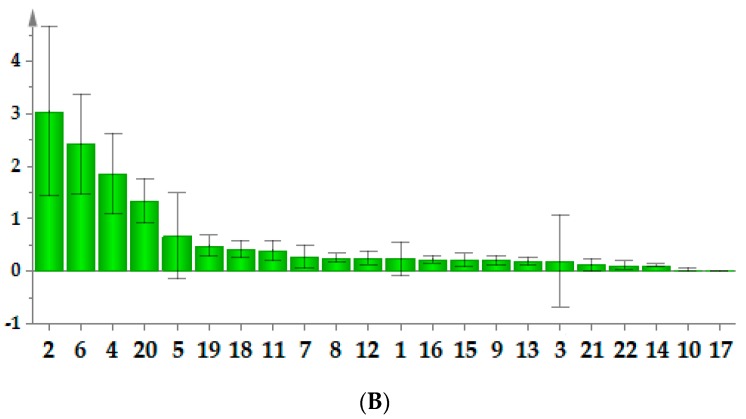
The orthogonal partial least squares discriminant analysis (OPLS–DA) scores scatter plot (**A**) and VIP (**B**) of xylem and phloem in SC.

OPLS–DA, a supervised latent structures–discriminant analysis method, utilizes class information to maximize the separation between classes and to minimize the separation between intra–groups. In order to further characterize the differences in secondary metabolites among the xylem and phloem, OPLS–DA was performed to obtain better discrimination among the two sections [17,22]. The scores plot of OPLS–DA demonstrated that all samples were clearly classified into two groups: xylem and phloem. Besides, to discover which chemical compositions contributed most to the clusters among the xylem and phloem, a VIP (variable importance for the projection) plot was constructed in the OPLS–DA model. The OPLS–DA scores scatter plot and VIP values were shown in Figure 5A,B. The established OPLS–DA model showed good adaptability (R^2^X = 0.953, R^2^Y = 0.878) and predictability (Q^2^ = 0.761). The results illustrated that the differences of constituents between xylem and phloem are remarkable. Based on their VIP values (i.e., larger than 1.0), 4 compounds were found to play key roles in the clusters, including protocatechuic acid (**2**), epicatechin (**6**), catechin (**4**) and medicarpin (**20**).

### 2.8. T–Test of Samples

**Figure 6:**
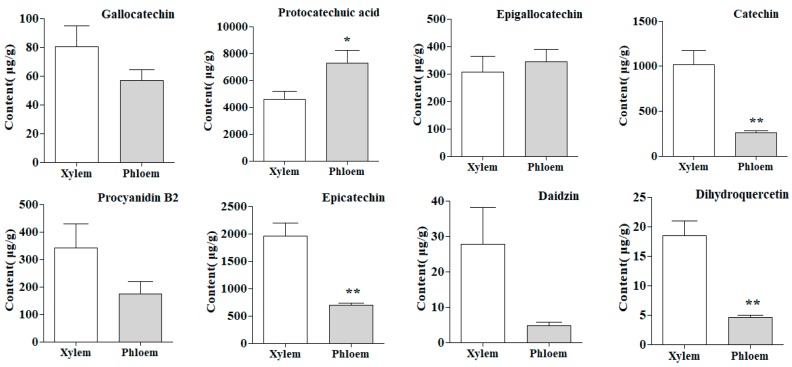

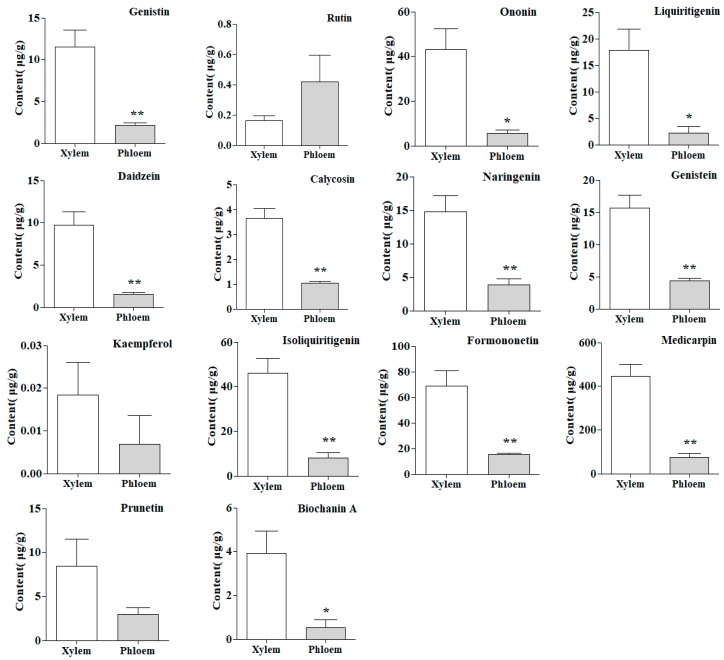
The average contents of 22 constituents in xylem and phloem of SC (* *p* < 0.05; ** *p* < 0.01).

In order to evaluate the content difference of 22 constituents in xylem and phloem, *t*-test analysis was used and the values of *p* less than 0.05 were considered remarkably different [20,21]. As illustrated in Figure 6, more than half of the bioactive constituents investigated in this study showed significant difference between xylem and phloem. Xylem contained a dramatically higher amount (*p* < 0.05) of catechin (**4**), epicatechin (**6**), dihydroquercetin (**8**), genistin (**9**), ononin (**11**), liquiritigenin (**12**), daidzein (**13**), calycosin (**14**), naringenin (**15**), genistein (**16**), isoliquiritigenin (**18**), formononetin (**19**), medicarpin (**20**) and biochanin A (**22**) than the phloem. However, phloem displayed remarkably higher content (*p* < 0.05) of protocatechuic acid (**2**). In the other compounds, the changes of contents in both xylem and phloem showed less difference.

## 3. Discussion

As mentioned previously, Spatholobi Caulis is a unique drug with high medicinal value [6]. The demand for SC in clinical application is increasing. In recent years, there have been few reports on the quality study of SC at home and abroad and the refinement research on the distribution of components in different parts is even rare. In this study, we sought to establish a methodology for exploring the distribution patterns of metabolites in xylem and phloem of SC. We found that the contents of flavonoids in the xylem were higher than that in the phloem and the content of protocatechuic acid exactly showed a contrary tendency, that is, the contents in the phloem were higher than that in the xylem. The scores scatter plot of PCA and OPLS–DA showed that the xylem and phloem samples were clearly divided into two groups (Figure 4 and Figure 5A). These findings elaborated the distribution laws of metabolites in xylem and phloem of SC, confirming that flavonoids are mainly distributed in xylem, while protocatechuic acid is mainly distributed in phloem, which helps to provide a scientific basis for revealing the accumulation rule of metabolites in SC. Traditionally, SC with deep reddish-brown and more resinous secretions on the cut surface is thought to be of better quality [10,11]. Zongwan Xie, a famous Chinese medicine expert, put forward the idea of “assessing the quality by distinguishing features of traditional Chinese medicinal materials” [23]. Pharmacological studies have shown that protocatechuic acid in SC possess obvious pharmacological activity on the cardiovascular system [9]. Flavonoids have the function of being anti-anemia [8]. We speculate that protocatechuic acid is related to the blood-activating effect of SC, while flavonoids are related to the blood-enriching effect. The medicinal materials with larger phloem may be better for activating blood, while the medicinal materials with more xylem may be better for enriching blood. It is just our guess. We will continue to explore and study further in the future. Most notably, this study could have quite a little reference significance for the quality formation of SC. The findings from this research could be employed as guidance in the market selection of SC.

## 4. Materials and Methods

### 4.1. Plant Materials

**Figure 7:**
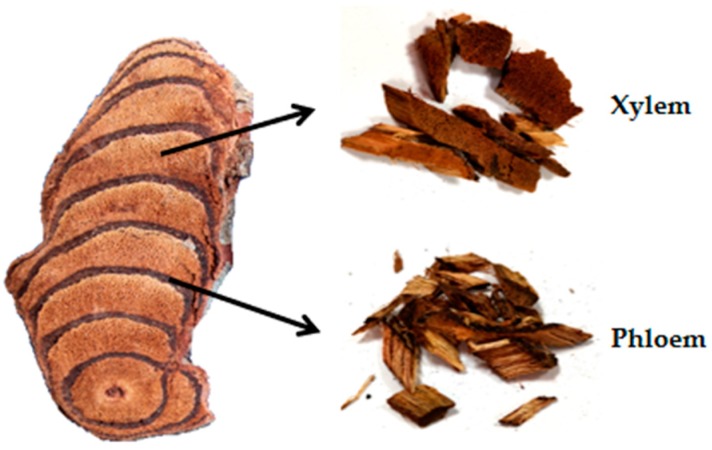
The xylem and phloem of Spatholobi Caulis.

S1–1, S2–1, S3–1, S4–1 and S5–1 were the xylem of SC, S1–2, S2–2, S3–2, S4–2 and S5–2 were the phloem of SC, which were all collected from Laos. All the samples were oval or irregular dry oblique sections with a thickness of about 0.3–1 cm. Each of them was carefully divided into xylem and phloem without sticking to each other, as shown in Figure 7. All the samples were authenticated by Professor Xunhong Liu (Nanjing University of Chinese Medicine, Nanjing, China) and were deposited in the laboratory of Chinese medicine identification, Nanjing University of Chinese Medicine.

### 4.2. Chemicals and Reagents

The standards of procyanidin B2 (**5**), daidzin (**7**), dihydroquercetin (**8**), genistin (**9**), liquiritigenin (**12**), calycosin (**14**), naringenin (**15**), genistein (**16**), medicarpin (**20**), prunetin (**21**) and biochanin A (**22**) were purchased from Nanjing Liangwei biotechnology Co., Ltd. (Nanjing, China). Epicatechin (**6**), rutin (**10**), kaempferol (**17**) and formononetin (**19**) were purchased from the Chinese National Institute for the Control of Pharmaceutical and Biological Products (Beijing, China). Ononin (**11**), gallocatechin (**1**), epigallocatechin (**3**) and isoliquiritigenin (**18**) were purchased from Chengdu Chroma Biotechnology Co., Ltd. (Chengdu, China). Daidzein (**13**) and protocatechuic acid (**2**) were purchased from Shanghai Yuanye Biotechnology Co., Ltd. (Shanghai, China). Catechin (**4**) was purchased from Baoji Chenguang Biotechnology Co., Ltd. (Baoji, China). The purities of all compounds were greater than 98%, checked by HPLC analysis. The structures of the 22 standards were shown in Figure S1. Formic acid, acetonitrile and methanol of HPLC grade were purchased from Merck (Darmstadt, Germany). Chloroform was HPLC pure and purchased from Shanghai Lingfeng Chemical Reagent Co., Ltd. (Shanghai, China). Ultrapure water was prepared using a Milli–Q water purification system (Millipore, Bedford, MA, USA).

### 4.3. Preparation of Standard Solutions

A mixed standard stock solution containing 22 reference substances was prepared with methanol at the following concentrations: 0.970 (**1**), 1.042 (**2**), 0.994 (**3**), 0.996 (**4**), 1.035 (**5**), 1.004 (**6**), 1.110 (**7**), 0.980 (**8**), 1.032 (**9**), 0.990 (**10**), 0.988 (**11**), 1.052 (**12**), 1.006 (**13**), 1.010 (**14**), 1.038 (**15**), 1.002 (**16**), 1.006 (**17**), 1.300 (**18**), 1.026 (**19**), 1.135 (**20**), 0.456 (**21**), 1.184 (**22**) mg/mL, then diluted with methanol to a series of different concentrations for creation of the calibration curves. All of the solutions were stored at 4 °C and filtered through the 0.22 µm membranes (Jinteng laboratory equipment Co., Ltd., Tianjin, China) before LC–MS analysis.

### 4.4. Preparation of Sample Solutions

Five batches of Spatholobi Caulis were collected to separate xylem and phloem and they were crushed and screened through a 50-mesh sieve. The sample powders of xylem and phloem (1.0 g) were accurately weighed and ultrasonically extracted with 10 mL chloroform–methanol (4:1, *v*/*v*) for 60 min, respectively. After cooling down to room temperature, chloroform-methanol (4:1, *v*/*v*) was added to compensate for the weight loss. The extract was then filtered and the filtrate was centrifuged at a speed of 12,000 r/min for 10 min. Afterwards, the supernatant was diluted 10 times and filtered through a 0.22 μm microporous membrane (Jinteng laboratory equipment Co., Ltd., Tianjing, China) and stored at 4 °C before LC–MS analysis.

### 4.5. Chromatographic and Mass Spectrometric Conditions

The samples of Spatholobi Caulis were analyzed by SIL–20A UFLC XR system (Shimadzu, Kyoto, Japan). The column used for chromatographic analysis was XBridge^®^C_18_ column (4.6 mm × 100 mm, 3.5 μm) at 30 °C with gradient elution of 0.3% formic acid aqueous solution (A)–methanol (B) and the flow rate was 0.8 mL/min. The injection volume was 2 μL and the elution gradient was optimized as follows: 0–5 min, 15–30% B; 5–10 min, 30–40% B; 10–13 min, 40–45% B; 13–15 min, 45% B; 15–18 min, 45–55% B; 18–23 min, 55–65% B; 23–28 min, 65–100% B; 28–30 min, 100%B.

The API5500 triple quadrupole linear ion trap tandem mass spectrometer (AB SCIEX, Framingham, MA, USA) was used to detect samples by the multiple reaction monitoring mode (MRM) under both positive and negative electrospray ionization mode (ESI). The mass spectrometry parameters were set as below: curtain gas (CUR) flow rate: 40 L/min; atomized gas (GS1) flow rate: 55 L/min; auxiliary gas (GS2) flow rate: 55 L/min; ionization temperature (TEM): 550 °C; spray voltage (IS): 4500 V in positive ion mode and −4500 V in negative ion mode.

### 4.6. Validation of the Method

The curve of peak area (*Y*) versus corresponding concentration (*X*, ng/mL) was drawn to obtain each standard calibration curve and the regression equation, correlation coefficient and linear range were calculated through the standard curve. Under the condition of signal–to–noise ratio (S/N) of 10 and 3, LOQ and LOD of 22 compounds were determined, respectively. Intra–day and inter–day precisions were determined with the standard solution for 6 times within a single day and 3 consecutive days for every analyte, respectively. The precision of the method was determined by the relative standard deviation (RSD) of the peak area. To test its repeatability, the same sample was parallelly divided in 6 parts and extracted by the same method and analyzed by UFLC–QTRAP–MS/MS as mentioned above. In order to examine the inherent stability characteristics of the tested compounds, the same sample was analyzed at different time intervals of 0 h, 2 h, 4 h, 8 h, 12 h and 24 h. A recovery test for assessment of accuracy was performed using the standards addition method. Three concentration levels of 22 authentic standards (approximately equivalent to 80%, 100% and 120% levels of each compound) were added into the same sample, respectively. Each set of addition was repeated three times and the spiked samples were analyzed by UFLC–QTRAP–MS/MS. The extraction recovery percentages were calculated by the following equation:

Recovery percentage (%) = (Amount determined − Amount original)/Amount spiked × 100%.(1)

Matrix effect refers to the phenomenon that the presence of substances other than the target analyte directly or indirectly affects the response of the analyte to be tested in the process of sample testing. Due to the high selectivity of mass spectrometry, the matrix effect is often invisible on the chromatogram but these co–eluting components can change the ionization efficiency of the substance to be measured, resulting in the inhibition or improvement of their detection signal [24]. In this study, the matrix effects were evaluated by the slope comparison method. Standard addition calibration curves were constructed by analyzing the sample extracts spiked with appropriate amounts of standards. Then the slopes of the standard addition calibration curves were compared with the slopes of the pure methanol standards. The slope ratio (slope matrix/slope solvent) was calculated to evaluate the matrix effect. If the ratio is less than 1.0, it indicates that the matrix has an inhibitory effect on the response of the analyte; if the ratio is greater than 1.0, it is opposite to the above; if it is equal to 1.0, the response of the analyte is unaffected, which is the most ideal situation and the highest goal when establishing the detection method [25].

### 4.7. Multivariate Statistical Analysis

According to the contents of 22 components, the xylem and phloem of SC were compared and analyzed by principal component analysis (PCA), which is an unsupervised pattern recognition method, with the software of Simca–P 13.0 (Umetrics AB, Umea, Sweden). In addition, OPLS–DA, a supervised latent structures–discriminant analysis method, was applied to disclose which chemical components contributed most to the clusters of xylem and phloem and a VIP map was obtained in the model. The content data of the 22 components tested, on the other hand, were statistically analyzed by *t*–test (SPSS 21.0, IBM SPSS, New York, NY, USA) to find the differential components of the xylem and phloem. Then according to the results of *t*-test analysis, the histograms of each component in the xylem and phloem were drawn with GraphPad Prism 5.0 software (Graphpad Software, San Diego, CA, USA), which could more clearly perceive the content distribution difference of different components in the xylem and phloem of SC.

## 5. Conclusions

In this experiment, UFLC–QTRAP–MS/MS was established for simultaneous quantification of 4 flavanols, 9 isoflavones, 2 flavonols, 2 dihydroflavones, 1 flavanonol, 1 chalcone, 1 pterocarpan, 1 anthocyanidin and 1 phenolic acid in different segments of SC and the contents of 22 constituents in the xylem and phloem of SC were compared and evaluated combined with multivariate statistical analysis. The results demonstrated that the contents of flavonoids in the xylem were higher than that in the phloem and the content of protocatechuic acid exactly presented the opposite trend. PCA and OPLA–DA based on the quantitative data elaborated the metabolites between xylem and phloem were significantly different and 4 different compounds (protocatechuic acid, epicatechin, catechin and medicarpin) were significantly related to sample classification. Moreover, the *t*–test results also showed that these compounds were remarkably different in the xylem and phloem. The distribution of secondary metabolites in xylem and phloem of SC was elucidated by comparing and analyzing these differences. More than that, the results could provide the basic foundation for announcing the laws of metabolite accumulation in SC. It is also more beneficial to further study the quality formation of SC.

## Figures and Tables

**Table 1:** Retention time, related mass spectrometer data of the standard compounds.

No.	Compounds	tR (min)	Precursor Ion (m/z)	Product Ion (m/z)	DP (V)	CE (eV)
1	Gallocatechin	2.30	307.10	139.00	100	13
2	Protocatechuic acid	3.39	152.90	109.00	−85	−18
3	Epigallocatechin	3.69	307.10	139.00	100	13
4	Catechin	4.06	291.10	139.00	90	13
5	Procyanidin B2	4.34	579.20	291.10	120	13
6	Epicatechin	5.93	291.10	139.00	90	13
7	Daidzin	7.24	417.70	256.60	32	23
8	Dihydroquercetin	8.24	305.10	153.10	105	15
9	Genistin	9.20	431.30	268.00	−105	−39
10	Rutin	10.74	610.93	302.94	30	18
11	Ononin	13.63	431.10	269.10	100	10
12	Liquiritigenin	13.75	257.10	137.10	125	15
13	Daidzein	14.94	255.10	199.10	155	29
14	Calycosin	16.24	285.10	270.00	135	25
15	Naringenin	17.23	273.00	153.00	120	25
16	Genistein	18.16	271.10	91.00	155	35
17	Kaempferol	19.56	257.00	137.10	−120	−36
18	Isoliquiritigenin	20.38	285.00	116.90	115	15
19	Formononetin	21.54	269.10	197.10	135	35
20	Medicarpin	22.02	271.47	137.08	116	19
21	Prunetin	23.79	285.10	192.00	165	30
22	Biochanin A	24.36	285.10	192.00	165	30

**Table 2:** Regression equation, limits of detection (LOD) and limits of quantification (LOQ), precision, repeatability, stability and recovery of 22 investigated compounds.

No.	Compounds	Regression Equation	*r* ^2^	Liner Range (ng/mL)	LOD (ng/mL)	LOQ (ng/mL)	Precision RSD (%)		Repeat-Ability RSD (%) (*n* = 6)	Stability RSD (%)(*n* = 6)	Recovery (%) (*n* = 9)						Matrix Effect
							Intra–Day (*n* = 6)	Inter–Day (*n* = 9)			Low		Medium		High		
											Mean	RSD (%)	Mean	RSD (%)	Mean	RSD (%)	
1	Gallocatechin	Y = 4000X − 102,000	0.9995	40.00–4000	2.03	6.75	3.46	3.47	4.42	3.71	99.37	0.97	100.80	1.18	98.17	3.26	1.03
2	Protocatechuic acid	Y = 63.3X + 233,000	0.9994	58.59–600,000	7.99	26.63	2.75	3.81	1.65	3.08	98.29	3.05	100.96	4.57	98.36	2.96	0.98
3	Epigallocatechin	Y = 2650X − 348,000	0.9999	164.82–16,500	4.31	14.36	0.93	2.79	3.71	3.35	98.51	3.14	97.90	1.41	98.21	1.08	0.95
4	Catechin	Y = 1990X + 330,000	0.9993	9.53–19,100	0.70	2.32	3.29	3.65	1.57	1.84	100.71	4.28	100.17	2.81	99.43	2.77	1.06
5	Procyanidin B2	Y = 506X + 21,000	0.9999	10.87–10,900	2.04	6.79	2.71	4.30	2.66	2.34	102.31	2.90	99.45	4.62	101.36	4.81	1.02
6	Epicatechin	Y = 1900X + 428,000	0.9996	3.13–31,300	0.82	2.74	2.43	3.09	2.46	4.24	100.16	2.46	102.87	0.96	100.22	1.57	1.05
7	Daidzin	Y = 221X − 236	0.9997	5.63–1408	0.94	3.13	3.48	3.76	2.03	2.68	100.27	0.76	100.30	2.90	99.24	2.44	1.00
8	Dihydroquercetin	Y = 494X + 2340	0.9997	10.20–511	1.02	3.40	4.19	2.22	3.03	2.44	101.22	2.85	103.93	1.13	97.99	2.02	1.04
9	Genistin	Y = 658X − 6	0.9997	1.26–630	0.27	0.90	2.68	1.86	1.69	0.73	103.83	0.95	101.18	4.41	100.77	4.94	0.96
10	Rutin	Y = 5300X + 3540	0.9990	0.37–36.90	0.09	0.28	3.29	3.55	1.08	4.16	100.73	1.56	102.37	2.45	99.52	1.32	0.99
11	Ononin	Y = 7620X + 77,700	0.9995	3.78–3785	0.79	2.63	2.55	4.16	1.99	1.90	101.99	2.02	101.28	1.17	99.93	4.46	1.04
12	Liquiritigenin	Y = 1420X + 18,500	0.9991	14.52–1450	2.59	8.65	3.31	2.95	3.67	4.11	96.69	2.08	102.00	1.95	100.32	2.28	1.04
13	Daidzein	Y = 8280X + 11,500	0.9997	1.54–772	0.3	1.00	4.20	3.53	4.23	2.60	99.38	4.19	98.76	2.81	101.80	2.16	1.02
14	Calycosin	Y = 12,700X − 15,000	0.9995	2.44–244	0.17	0.58	1.08	3.62	2.77	3.52	95.88	1.20	97.98	3.01	100.71	3.87	0.94
15	Naringenin	Y = 6160X + 91,100	0.9996	6.87–1370	1.04	3.47	1.91	3.86	3.39	1.16	96.91	2.78	99.66	4.24	99.96	4.26	0.99
16	Genistein	Y = 1280X + 6000	0.9996	15.30–611	1.87	6.24	2.84	3.38	0.76	2.99	102.17	1.92	103.48	1.20	102.50	1.50	0.97
17	Kaempferol	Y = 1630X + 194	0.9994	0.28–11.10	0.07	0.22	1.99	2.02	2.83	2.34	103.68	0.91	103.21	1.38	102.22	1.49	1.02
18	Isoliquiritigenin	Y = 961X + 15,700	0.9998	31.59–1260	4.12	13.73	3.02	3.55	1.27	1.09	103.35	1.01	101.06	3.15	101.91	2.11	1.00
19	Formononetin	Y = 2530X + 71,300	0.9992	9.51–3800	1.95	6.51	1.46	3.62	1.01	1.39	103.22	2.04	102.84	1.06	99.17	3.65	0.93
20	Medicarpin	Y = 2030X + 76,100	0.9995	19.29–19,292	3.33	11.09	1.03	1.88	3.72	3.78	101.02	4.78	97.78	3.97	101.09	3.15	0.98
21	Prunetin	Y = 10.9X + 229	0.9990	5.80–232	1.39	4.64	2.23	1.75	2.23	2.70	97.95	3.19	102.44	3.10	101.39	4.73	1.00
22	Biochanin A	Y = 21.3X + 131	0.9995	8.83–177	0.76	2.52	3.54	2.60	2.39	2.15	101.03	0.99	100.58	2.61	102.47	2.22	1.08

**Table 3:** Contents of 22 compounds in Spatholobi Caulis (μg/g, *n* = 3).

No.	Compounds	Xylem					Phloem				
		S1–1	S2–1	S3–1	S4–1	S5–1	S1–2	S2–2	S3–2	S4–2	S5–2
1	Gallocatechin	96.8	121.06	88.06	52.32	43.80	63.53	70.29	72.01	39.30	39.32
2	Protocatechuic acid	5745.66	4166.29	5161.65	5352.32	2586.10	10118.61	4607.29	8048.11	7515.76	6127.87
3	Epigallocatechin	369.74	488.65	296.56	216.59	159.92	450.73	377.96	416.70	225.61	253.64
4	Catechin	1214.57	1300.13	1244.85	853.02	483.92	293.38	210.51	306.38	270.38	217.70
5	Procyanidin B2	399.01	559.15	456.37	229.12	64.43	258.62	176.45	284.25	94.48	55.76
6	Epicatechin	2124.84	2519.83	2177.69	1883.30	1088.00	766.72	619.46	808.65	677.54	583.03
7	Daidzin	35.90	63.91	20.34	7.12	11.74	7.53	7.21	2.32	2.39	3.91
8	Dihydroquercetin	17.34	26.25	20.78	16.62	11.45	4.42	3.59	4.02	5.40	5.34
9	Genistin	14.58	15.81	12.80	9.88	4.53	3.18	2.22	1.73	1.78	1.92
10	Rutin	0.26	0.15	0.20	0.08	0.12	1.01	0.28	0.06	0.62	0.13
11	Ononin	69.19	60.14	38.62	21.43	26.28	11.47	4.92	2.93	3.22	5.27
12	Liquiritigenin	25.18	21.02	14.12	3.74	25.11	2.17	–	1.43	–	7.15
13	Daidzein	12.06	14.72	7.63	8.11	6.29	2.05	1.46	0.80	1.29	2.12
14	Calycosin	3.76	5.06	3.21	3.57	2.61	0.89	0.98	0.94	1.05	1.32
15	Naringenin	19.95	11.96	14.42	7.24	20.11	4.20	1.95	2.22	3.72	7.00
16	Genistein	19.14	21.64	11.95	12.89	12.73	4.12	3.99	3.31	4.36	6.01
17	Kaempferol	0.03	0.03	0.03	–	–	–	0.03	–	–	–
18	Isoliquiritigenin	59.97	51.23	41.14	22.93	55.29	8.77	4.64	4.08	5.54	17.00
19	Formononetin	92.83	84.15	82.57	28.73	56.87	15.52	16.63	12.08	14.50	18.50
20	Medicarpin	557.83	597.30	371.66	353.01	352.41	93.27	58.31	38.50	45.52	136.22
21	Prunetin	18.08	12.95	6.29	3.63	1.18	1.79	5.61	3.25	1.38	3.02
22	Biochanin A	7.51	3.90	3.49	1.17	3.59	1.69	–	–	–	0.97

Note: “–”not detected.

## Supplementary Materials

The following are available online. Figure S1: Chemical structures of 22 reference substances.

## References

[1] Chinese Pharmacopoeia Committee (2015). Pharmacopoeia of the People’s Republic of China. Part I.

[2] Fu Y., Cheng Y., Chen J.P., Wang D.M. (2011). Advances in Studies on Chemical Constituents in Spatholobi Caulis and their Pharmacological Activities. Chin. Tradit. Herbal. Drugs..

[3] Chen X., Li Q., Kan X.X., Wang Y.J., Li Y.J., Yang Q., Xiao H.B., Chen Y., Weng X.G., Cai W.Y. (2016). Extract of *Caulis Spatholobi*, a Novel Blocker Targeting Tumor Cell-Induced Platelet Aggregation, Inhibits Breast Cancer Metastasis. Oncol. Rep..

[4] Liu B., Liu J.L., Chen J., Zhu D.M., Zhou H.J., Wang X.M. (2013). A Study on Anticancer Activity of Caulis Spatholobi Extract on Human Osteosarcoma Saos-2 cells. Afr. J. Tradit. Complement. Altern. Med..

[5] Cao Y.N., Tang Q.L., Luo L., Gao Z.Y., Zhang S.Z. (2018). A Preliminary Study on the Anticancer Efficacy of Caulis Spatholobi Compound 1802. Pak. J. Pharm. Sci..

[6] Qin S.S., Zhu Y.X., Wei K.H., Li M.J., Miao J.H., Zhang Z.Y. (2018). Study on Herbal Textual Evolution and Flavonoids and their Pharmacological of Spatholobi Caulis. China J. Chin. Mater. Med..

[7] Huang S.Y., Luo J.H., Zhang L.D., Meng C.Y., Feng L., He Q.Z. (2007). Extraction of Total Flavanone from *Spatholobus suberctus* Dunn and its Effects on Scavenging of Free Radicals. Lishizhen Med. Mater. Med. Res..

[8] Liang N., Wei S.J., Lin Q.Y. (2009). Study on the Blood Enriching Effect and Mechanism of Spatholobus Suberect Total Flavonoids. Lishizhen Med. Mater. Med. Res..

[9] Zhai M., Liu J.M., An R., Wu F.L., Xu H.H. (2009). Content Determination of Protocatechuic Acid in *Spatholobus suberctus* Dunn. Tradit. Chin. Drug. Res. Clin. Pharmacol..

[10] State Administration of Traditional Chinese Medicine (1999). Chinese Materia Medica. Part 4.

[11] Yao R.L., Rong Y.W. (2017). Identification Technology of Traditional Chinese Medicine.

[12] Wu W.Y., Guo D.A. (2014). Strategies for Elaboration of Comprehensive Quality Standard System on Traditional Chinese Medicine. China J. Chin. Mater. Med..

[13] Lu X.L., Pan X.J., Deng M.M., Wu J.X., Zhao J.H. (2018). TLC Identification and Determination of Catechin and Epicatechin in Spatholobi Caulis Extract. Chin. J. Exp. Tradit. Med. Form..

[14] Liang Y.S., An R., Liu J.M., Huang Y.Y. (2013). Determination of Genistein and Formononetin in Spatholobi Caulis from Different Habitats. Lishizhen Med. Mater. Med. Res..

[15] Huang C.H. (2009). Determination of Dihydroxy Benzoic Acid in Suberect Spatholobus of Different Place of Production with HPLC Method. Guid. J. Tradit. Chin. Med. Pharm..

[16] Zhang Y., Guo L., Duan L., Dong X., Zhou P., Liu E.H., Li P. (2015). Simultaneous Determination of 16 Phenolic Constituents in Spatholobi Caulis by High Performance Liquid Chromatography/Electrosprayionization Triple Quadrupole Mass Spectrometry. J. Pharm. Biomed. Anal..

[17] Zhu L.X., Xu J., Zhang S.J., Wang R.J., Huang Q., Chen H.B., Dong X.P., Zhao Z.Z. (2018). Qualitatively and Quantitatively Comparing Secondary Metabolites in Three Medicinal Parts Derived from *Poria cocos* (Schw.) Wolf using UHPLC-QTOF-MS/MS-Based Chemical Profiling. J. Pharm. Biomed. Anal..

[18] Du Z.X., Li J.H., Zhang X., Pei J., Huang L.F. (2018). An Integrated LC-MS-Based Strategy for the Quality Assessment and Discrimination of three *Panax* Species. Molecules.

[19] Chen L., Tang Z.S., Song Z.X., Liu Y.R., Hu J.H., Shi X.B., Sun C., Jiang D.H., Li X.H. (2019). Quantitative Determination of Nine Furanocoumarins for Quality Evaluation of *Angelica dahurica* from Different Habitats. China J. Chin. Mater. Med..

[20] Chen S.Y., Shi J.J., Zou L.S., Liu X.H., Tang R.M., Ma J.M., Wang C.C., Tan M.X., Chen J.L. (2019). Quality Evaluation of Wild and Cultivated *Schisandrae chinensis* Fructus Based on Simultaneous Determination of Multiple Bioactive Constituents Combined with Multivariate Statistical Analysis. Molecules.

[21] Tan M.X., Chen J.L., Wang C.C., Zou L.S., Chen S.Y., Shi J.J., Mei Y.Q., Wei L.F., Liu X.H. (2019). Quality Evaluation of Ophiopogonis Radix from two Different Producing Areas. Molecules.

[22] Senizza B., Rocchetti G., Ghisoni S., Busconi M., De Los Mozos Pascual M., Fernandez J.A., Lucini L., Trevisan M. (2019). Identification of Phenolic Markers for Saffron Authenticity and Origin: An Untargeted Metabolomics Approach. Food. Res. Int..

[23] Peng H.S., Zhang H.T., Peng D.Y., Cheng M.E., Zha L.P., Huang L.Q. (2017). Evolution and Characteristics of System, Assessing Quality by Distinguishing Features of Traditional Chinese Medicinal Materials, of Dao-Di Herbs of Astragali Radix. China J. Chin. Mater. Med..

[24] Wang P., Jiang X.H., Wang L. (2011). Assessment of Matrix Effect in Quantitative Bioanalytical Methods Based on LC-MS^n^. Chin. New Drugs J..

[25] Wang L.Q., He L.M., Zeng Z.L., Chen J.X. (2011). Progress in Matrix Effect of Veterinary Drug Residues Analysis by High-Performance Liquid Chromatography Tandem Mass Spectrometry. J. Mass. Spectrom..

