# Supplementary material for: A Method to Study the Distribution Patterns for Metabolites in Xylem and Phloem of Spatholobi Caulis
Source: Molecules. 2019 Dec 31;25(1):167. doi: 10.3390/molecules25010167 (PMC6983255; doi:10.3390/molecules25010167)
Supplement: Supplementary file 1 [file molecules-25-00167-s001.pdf]

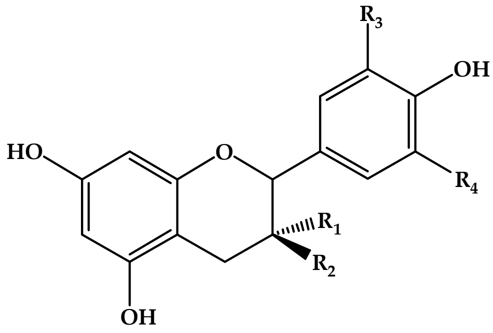

No.	Compound	R1	R2	R3	R4
1	Gallocatechin	H	OH	OH	OH
3	Epigallocatechin	OH	H	OH	OH
4	Catechin	H	OH	H	OH
6	Epicatechin	OH	H	H	OH

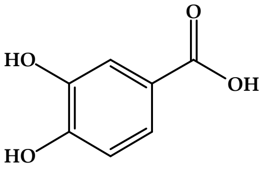

2 Protocatechuic acid

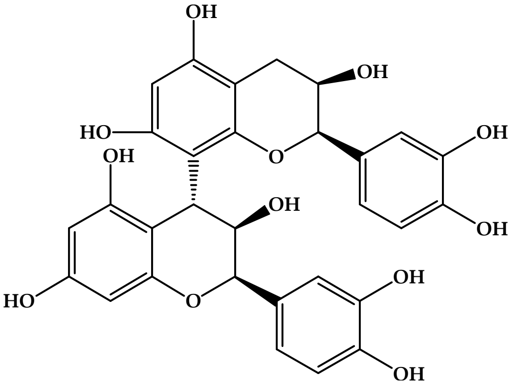

5 Procyanidin B2

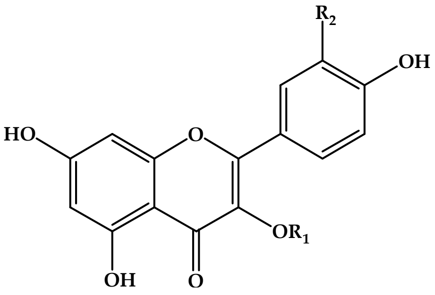

No.	Compound	R1	R2
10	Rutin	rutinose	OH
17	Kaempferol	H	H

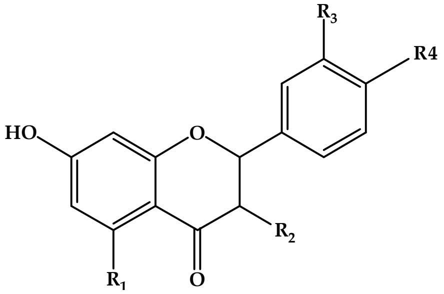

No.	Compound	R1	R2	R3	R4
8	Dihydroquercetin	OH	OH	OH	OH
12	Liquiritigenin	H	H	H	OH
15	Naringenin	OH	H	H	OH

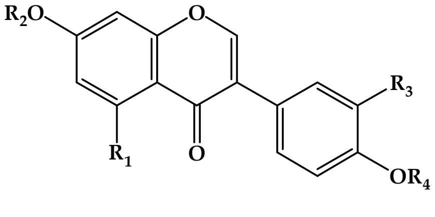

No.	Compound	R1	R2	R3	R4
7	Daidzin	H	glc	H	H
9	Genistin	OH	glc	H	H

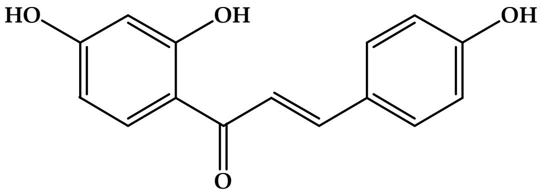

18 Isoliquiritigenin

11	Ononin	H	glc	H	CH_3
13	Daidzein	H	H	H	H
14	Calycosin	H	H	OH	CH_3
16	Genistein	OH	H	H	H
19	Formononetin	H	H	H	CH_3
21	Prunetin	OH	CH_3	H	H
22	Biochanin A	OH	H	H	CH_3

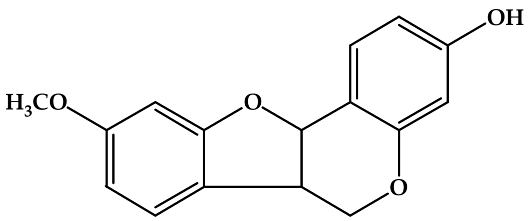

20 Medicarpin

Figure S1. Chemical structures of 22 reference substances.
